# A rat model of early stage osteonecrosis induced by glucocorticoids

**DOI:** 10.1186/1749-799X-6-62

**Published:** 2011-12-21

**Authors:** Mohammad Amin Kerachian, Edward J Harvey, Denis Cournoyer, Terry Y Chow, Ayoub Nahal, Chantal Séguin

**Affiliations:** 1 Department of Human Genetics, McGill University Health Center (MUHC), 1650 Cedar Avenue, Montreal, QC H3G 1A4, Canada; 2 Department of Medical Genetics, Mashhad University of Medical Sciences (MUMS), Azadi Square, Mashhad, 917794-8564, Iran; 3 Division of Orthopaedic Surgery, McGill University Health Center (MUHC), 1650 Cedar Avenue, Montreal, QC H3G 1A4, Canada; 4 Department of Medicine, Division of Haematology, McGill University Health Center (MUHC), 1650 Cedar Avenue, Montreal, QC H3G 1A4, Canada; 5 Department of Oncology, McGill University Health Center (MUHC), 1650 Cedar Avenue, Montreal, QC H3G 1A4, Canada; 6 Department of Pathology, McGill University Health Center (MUHC), 1650 Cedar Avenue, Montreal, QC H3G 1A4, Canada

## Abstract

**Background:**

Glucocorticoid (GC)-induced osteonecrosis (ON) is an important complication of medical therapy. The exact pathomechanisms of ON has not been clearly elucidated. There is a need for a reproducible animal model that better approximates the clinical scenario.

**Methods:**

To determine the genetic susceptibility of rats to develop GC-induced femoral head ON, we evaluated 5 different inbred strains of rats (Spontaneous Hypertensive Rat, Wistar Kyoto, Wistar Furth, SASCO Fisher and Lewis). Prednisone pellets (dosage of 1.82-2.56 mg/kg/day) were implanted subcutaneously for 90. After 90 days, the femurs were resected and examined histologically and radiographically. Pathological and histological examination was performed. Hematoxylin and eosin (H & E) staining was used to delineate the femoral head osteonecrosis lesions as well as abnormalities of articular cartilage and growth plate.

**Results:**

The greatest differences in H & E staining were seen in the Wistar Kyoto and Wistar Furth groups. In these groups 4 out of 5 and 3 out of 5, respectively, steroid-induced rats revealed growth plate disruption with acellular areas. The TUNEL apoptosis staining assay for apoptosis revealed that 4 out of 5 of Wistar Kyoto rats, 5 out of 5 of Wistar Furth, 2 out of 4 of surviving Lewis and 2 out of 2 of the surviving spontaneous hypertensive rats had apoptotic osteocytes in trabeculae, whereas none of the Fisher rats showed apoptotic osteocytes.

**Conclusions:**

We postulate that Wistar Kyoto, Wistar Furth and spontaneous hypertensive rats may be strains of rats more susceptible to develop ON of the femoral head while Fisher rats were the most resistant.

## Background

Glucocorticoids (GCs) are widely prescribed in cases of rheumatoid arthritis, asthma, systemic lupus erythematosus, cancer, organ transplantation and many other medical conditions. The therapeutic use of GCs has been accompanied by marked side effects, especially with the long-term usage of this drug. The adverse effect of GCs on bone has been recognized for more than 60 years [1-3]. The bone effect is characterized by decreased bone formation and *in situ *death of isolated segments of bone which may be associated with osteonecrosis (ON) particularly important clinically for the femoral head. ON in the femoral head gradually progresses to fracture of the subchondral bone, collapse of the surface and hip arthritis. Although ON has been linked to a variety of conditions, GC usage remains the predisposing factor most commonly associated with the development of non-traumatic ON. There is considerable interest in identifying which patients are at highest risk for ON, with the long-term goal of modifying regimens to decrease the risk of adverse effects of therapy. Despite the strong association of GC administration with ON, the role of potential underlying risk factors such as hyperlipidemia, thrombophilia, and hyperfibrinolysis in the circulatory system remain unclear [2,4]. It has been clearly established that among patients receiving a specific dose of GC, only an unpredictable subset will develop ON. This underscores the existence of individual variability in the action of GCs and the potential presence of additional mechanisms and/or risk factors such as a genetic predisposition. On the other hand, studying the clinical pathology of ON in the early disease stage (before radiographic findings) is extremely difficult in human subjects. Thus, animal experiments are needed to elucidate the pathophysiology of the disorder. Having a suitable animal model would allow for the systemic evaluation of host-related (ie. genetic variations) as well as acquired (ie. treatment-related) risk factors. GC-induced ON has been induced in rabbit models [5-8], bipedal animals (e.g., chickens, emus) [9,10] and recently, in BALB/cJ mice [11]. GC-induced ON has been described in mature Japonese white rabbits (Kbs-JW) [5,8] but the genome of rabbit has only been incompletely sequenced, thus limiting the usefulness of that model for the identification of genes affecting the risk of developing ON. The biped models are difficult to interpret in the context of bone healing as we do not have a full grasp of avian bone healing. Although a mouse model of GC-induced ON is interesting, the very small diameter of the femoral head of mice limits the application of numerous experiments and monitoring techniques. It is currently impossible to read an MRI or radiograph from a mouse with the goal of differentiating a normal hip from a hip with ON changes. A rat model would allow easier radiographic interpretation, allow facile surgical interventions, allow existing small animal facilities to be used as well as be in an animal where the genetics of healing is much better understood. To date, there has been no rat model of GC-induced ON unless it has been combined with a surgical procedure [12] or in combination with immune responses stimuli [13]. These blood interruption studies do not faithfully model the more prevalent non-traumatic ON. In this study, our goal was to establish a rat model of GC-induced ON by screening different strains of rats in order to uncover those whose constitutive phenotype might predispose to the development of ON.

## Methods

### Maintenance and experimental animals

In this pilot study, female retired breeder (aged 6-8 months) Fisher, Lewis, Spontaneous Hypertensive, Wistar Kyoto, and Wistar Furth rats (6 of each strain) were obtained from Charles River Laboratories (Pointe-Claire, QC, Canada). The rats were tagged and housed in plastic cages (2 animals per cage) under standard laboratory conditions with a 12-hour dark/12-hour light cycle, a constant temperature of 20°C, and humidity of 48%. Food and water were provided ad libitum with a standard rodent diet. The weight of the rats were followed before and after the implant of a prednisone pellet for the first 4 consecutive days, then every week until the end of the experiment. All experiments were conducted under an animal protocol (Protocol No. 4935) approved by the McGill Animal Care Department, Montreal, Canada.

### Glucocorticoid administration

Slow-release prednisone pellets (Innovative Research of America, Sarasota, Florid, USA) were implanted subcutaneously in 5 inbred rats composing each group (Fisher, Lewis, Spontaneous Hypertensive, Wistar Kyoto and Wistar Furth). Each pellet was implanted underneath the skin on the lateral side of the neck by surgically making an incision and developing a pocket about 2 cm beyond the incision site. The pellet was placed in the pocket and the incision was sutured. Based on the manufacturer's instructions the pellet releases a constant dose of the drug subcutaneously. The average dose release from the pellet was equivalent to 1.82-2.56 mg/kg/day (mean: 2.26, SD: 0.19) for a period of 90 days. This dosage is an equivalent dosage to humans that commonly causes ON changes. Thus, each group had 5 GC-induced rats along with 1 control rat in each group not treated with prednisone (the control rat did not receive a placebo pellet).

### Histological Examination

The rats were sacrificed with an overdose of ketamine/xylazine following 90 days of the experiment. Tissue samples were obtained from the femoral head. Bone samples were fixed in 10% neutral buffered formalin overnight, then decalcified in 4% ethylenediamine tetraacetic acid (pH 7.2) (Sigma-Aldrich, St. Louis, MO, USA). The specimens were processed routinely and embedded in paraffin. Tissue sections were cut parasagitally with a rotary microtome to obtain 4 to 5 microns thickness, stained with hematoxylin and eosin (H & E) and evaluated by light microscopy.

Tissue samples were analyzed in a blinded fashion by an experienced bone pathologist (AN). GC-induced ON was diagnosed based on bone and growth plate changes. The histological findings of an established ON were defined as dead trabeculae exhibiting empty lacunae with or without appositional bone formation [14]. Occasional empty lacunae possibly created by sectioning through the edge of a lacunae was not considered as a sign for ON. The growth plate changes were considered as thinning, discontinuity pattern and disruption of articular cartilage alignment or growth plate alignment.

Tissue sections were also examined according to the criteria of Arlet *et al. *namely presence of degeneration, necrosis, and disappearance of marrow cells as well as the nuclear disappearance and hypochromasia of trabecular osteocytes as early signs of ON [15]. Early signs of ON was also considered when apoptosis occurred in the osteocytes and osteoblasts. Positivity for apoptosis was defined by the authors as 2 to 3 apoptotic osteocytes and/or osteoblasts considered as one plus, between 3 to 6 as two plus and more than 6 as three plus recognized in a high magnification field (×200). The experiments were performed in triplicate.

### Measurement of apoptosis in undecalcified bone section

We used terminal deoxynucleotidyl transferase dUTP nick end labeling (TUNEL assay) to detect DNA fragmentation by labeling the terminal end of nucleic acids. *In Situ *Cell Death Detection Kit was obtained from Roche (Germany). TUNEL assay on paraffin-embedded tissue sections was performed as recommended by the manufacturer. Briefly, after deparaffinization and permeabilization of the tissue sections with proteinase K, the slides were incubated with the TUNEL reaction mixture containing TUNEL-Enzyme solution and TUNEL-Label solution for 1 hour at 37°C inside a humidified chamber. After washing steps, samples were analyzed under a fluorescence microscope (in a drop of 1× PBS). The excitation wavelength ranged between 450-500 nm whereas the detection wavelength ranged between 515-565 nm (green). DNase I-treated tissue section was used as a positive control. Negative controls for the study constituted of sample slides processed using the same procedure but only treated with TUNEL-Label solution.

### Faxitron X-ray

Based on the histological results Faxitron x-ray analysis was performed initially on a group of Wistar Kyoto rats (5 rats, 10 femoral head samples) (Model MX-20). Previous work has shown that radiographic changes were a late finding in steroid induced ON in the rat model used. We performed the Faxitron radiographs on this group to ensure there were no significant changes despite changes on histology.

### Statistical Analysis

Comparison between groups was made with Fisher's Exact test. Significant differences were considered at P values less than 0.05.

## Results

We observed a high mortality rate in some strains of rats after prednisone implantation. Interestingly, the Lewis and spontaneous hypertensive strains of rats seemed to be at highest risk (mortality rate was 1 out of 5 and 3 out of 5, respectively and no mortality for other strains). There was an overall mortality rate of 16% among the steroid-treated rats in our pilot study related to the development of GC-induced hyperglycemia in these "older" rats (a two to three times fold increase compare to control rats).

**Figure 1:**
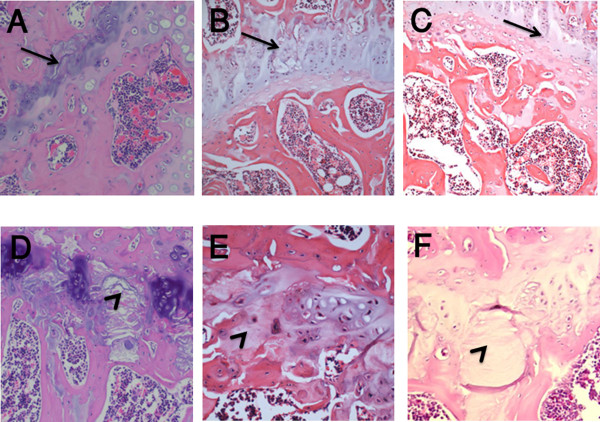
**Photomicrographs showing histological findings in steroid-induced rat models**. H & E staining in a control rat (A) versus glucocorticoid-induced rats of all groups (B: Lewis, C: Fisher, D: Wistar Kyoto, E: Wistar Furth and F: Spontaneous Hypertensive rats). Normal growth plate and its discontinuity pattern are shown by arrows and arrowheads, respectively. Magnification ×200.

**Figure 2:**
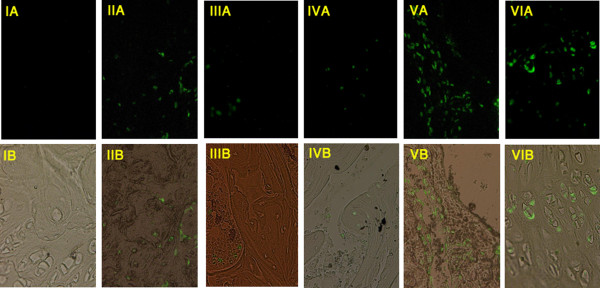
**Photomicrographs showing apoptosis of osteocytes as a marker of early osteonecrosis of the femoral head**. TUNEL staining apoptosis assay analyzed by (A): florescence microscope, (B): superimposed florescence and optical view on femoral head in II: Lewis, III: Fisher, IV: Wistar Kyoto, V: Wistar Furth and VI: Spontaneous Hypertensive rats induced with steroids for 3 months versus a control sample (I). The nucleus of apoptotic cells are shown in green. Magnification ×200.

Growth plate changes were observed in Wistar Kyoto and Wistar Furth rats (Figure 1). In these groups 4 out of 5 and 3 out of 5 of steroid-induced rats revealed growth plate disruption with acellular areas, respectively. Osteocyte necrosis and empty lacunae were not detected in any samples. TUNEL assay for apoptosis revealed that 4/5 of Wistar Kyoto, 5/5 of Wistar Furth, 2/4 of Lewis and 2/2 Spontaneous Hypertensive rats had apoptotic osteocytes in trabeculae, whereas none of the Fisher rats showed apoptotic osteocytes (Table 1, Figure 2). In the Lewis group, apoptosis of osteocytes and osteoblasts without any degeneration of the growth plate was observed. Overall, the most apoptosis rate was in spontaneous hypertensive rats (+++) and then Wistar Furth (++) and Wistar Kyoto (++). The apoptosis level in Lewis and Fisher rats was (+) and zero, respectively. Bone marrow and chondrocyte apoptotic cells were seen in all strains of rats, even the control rats as expected. There were no signs of inflammation and necrosis, such as hyperemia, round cell infiltration, or lipid cyst formation. Plain x-rays obtained from Faxitron analysis did not reveal any significant anomaly in the initial group of Wystar Kyoto rats. Often diminished bone density was noticed in rats exposed to glucocorticoid. The radiographs were not performed in the other groups because of the lack of changes. If there had been changes in the initial group the other strains would have been tested. This finding also confirmed that plain x-rays are not a suitable method to diagnose early stages of ON in rats.

**Table 1:** Apoptosis of the femoral head of GC-induced inbred rats from 5 different strains (WKY: Wistar Kyoto, WF: Wistar Furth, SHR: Spontaneous Hypertensive rat,).

Strain	Bone Marrow	Osteocyte	Chondrocyte
*Lewis*	4/4	2/4*	4/4
Fischer	5/5	0/5*	5/5
WKY	5/5	4/5*	5/5
WF	5/5	5/5*	5/5
*SHR*	2/2	2/2*	2/2

*Fisher's Exact Test (P value = 0.0039).

## Discussion

A strain dependant genetic predisposition may be responsible for the high mortality rate observed in some strains of rats after prednisone implantation. Recognition of this complication of hyperglycemia has been reported in young rats [16] and seems to be important when choosing a model for ON. It would make these two strains less desirable for this usage.

The rat growth plate is present throughout the life-cycle. This may confound the findings of ON for late stage ON in that reparative changes will overcome the initiating stimulus- particularly in traumatic vascular interruption studies. For this GC study, the high dosages of steroid given will result in early ongoing changes in the rat femoral head despite reparative processes from the growth plate. Because of this a rodent model is possible for early ON.

Growth plate disruption was observed particularly in Wistar Kyoto and Wister Furth strains. This was observed in early stages of the disease- before radiographic change was evident. Other studies using blood supply interruption (ischemic model) have also shown growth plate changes. Trueta and Amato used animal models and showed that the blood supply to the cartilage of the growth plate of the femoral head originates from the epiphyseal vessels [17], while the metaphysis is supplied by metaphyseal vessels and nutrient arteries coming from the medullary cavity. Mechanical damage to the metaphyseal arteries leads to destruction of the growth plate and, eventually, a physeal bridge [18]. It is possible that thrombosis in the metaphyseal arteries reported in ON of the femoral head could cause injury and disruption of the growth plate with areas lacking normal cells. Sato et al. have also shown that apoptosis tended to occur in early stages of ON. In their rat study of ischemic ON, apoptosis occurred 12 hours after the mechanical insult, whereas no evidence of apoptosis remained after 96 hours, at which time only empty lacunae were detected [19]. They postulated that the mechanism of cell death involved in ischemic ON was apoptosis as indicated by DNA fragmentation and the presence of apoptosis bodies in osteocytes [11,20]. In the present study, apoptosis of chrondrocytes were not only detected in GC-induced rats but also in control rats indicating that apoptosis of chondrocytes is not characteristic of ON but probably more indicative of normal bone turnover if observed in small amounts. Other studies have shown that apoptosis of osteocytes and osteoblasts is an important process in developing ON, especially in the early stages of ON [21]. Kabata and his colleagues demonstrated extensive osteocyte apoptosis in a rabbit model of GC-induced ON [22]. Shibahara et al. also reported the presence of a large number of apoptotic osteocytes around necrotic areas [23]. In the present study, we observed that apoptosis occurred at the level of osteocytes, osteoblasts, and bone marrow cells in the early stages of GC-induced ON lesions in three strains of inbred rats: Wistar Kyoto, Wistar Furth and Spontaneous Hypertensive. Apoptosis has been shown to play an important role in maintaining haematopoietic stem cells (HSC) homeostasis in *in vivo*. Thus, apoptosis of the HSC could occur as part of the normal physiology in bone marrow cells [24]. Previously, it has been shown that apoptosis could happen in bone marrow of control rats [25]. Apoptosis could result from a direct effect of GCs on the bone cells or could be secondary to the dysfunction/activation of other cells such as the femoral head endothelial cells [26]. Fisher rats were resistant to the development of osteocyte apoptosis in response to GC induction. Given the observed inter-strain variability of susceptibility to the development of GC-induced ON lesions, it is probable that genetic factors are involved in ON developing in response to GCs. To date different genetic variations and mutations accounting for ON have been reported. A 4G/4G mutation of the plasminogen activator inhibitor-1 gene [27], a G-- > A transition in exon 50 (p.G1170S) of collagen type II (COL2A1) [28] and a promoter polymorphisms of the vascular endothelial growth factor (VEGF) gene [29] were reported to be correlated with the occurrence of ON of the femoral head. Identifying the genetic factors may prove relevant to the human disorder and facilitate the identification of individuals at increased risk of developing ON.

## Conclusions

Based on these findings, Wistar Kyoto, Wistar Furth and Spontaneously Hypertensive rats were the most susceptible strains to develop GC-induced ON of the femoral head. The Spontaneously Hypertensive rats had a high mortality rate that is unacceptable for a study model. Fisher rats were resistant to the development of ON at the GC dosage used, based on the absence of osteocyte apoptosis in early stages of the disease process. Although several other investigations have reported ON in rats following the administration of GCs, our rat model has shown early stage disease regardless of additional adjuvants such as surgery [12] or immune response stimuli [13,30] as previously reported. It is possible that extended exposure to GCs could establish the histological criteria of the later stages of ON.

## Competing interests

The authors declare that they have no competing interests.

## Authors' contributions

All authors participated in the study. MAK made a major contribution to the writing of the manuscript's first draft, and conducted the experiments involved in the study. CS made a major contribution to the design of the study, data interpretation and scientific revision of the manuscript. DC, EJH and TYC made equal contributions to data interpretation and scientific revision of the manuscript. EJH made a major contribution to the editing and grammar of the manuscript. AN made major contributions to the histological experiments involved in the study. All authors participated in the manuscript preparation and revision. All authors read and approved the final manuscript.
